# Biodiversity and phylogeny of novel *Trichoderma* isolates from mangrove sediments and potential of biocontrol against *Fusarium* strains

**DOI:** 10.1186/s12934-019-1108-y

**Published:** 2019-05-23

**Authors:** Patrícia Rego Barros Filizola, Marcos Antônio Cavalcanti Luna, Adriana Ferreira de Souza, Iwanne Lima Coelho, Delson Laranjeira, Galba Maria Campos-Takaki

**Affiliations:** 1 Northeast Network for Biotechnology Post-graduation Program, Federal Rural University of Pernambuco, Recife, Pernambuco 52171-900 Brazil; 2 Federal Rural University of Pernambuco, Rua Dom Manoel de Medeiros, s/n, Dois Irmãos, Recife, Pernambuco 52171-900 Brazil; 3 Nucleus of Research in Environmental Sciences and Biotechnology, Catholic University of Pernambuco, Recife, Pernambuco 50050-590 Brazil

**Keywords:** Filamentous fungi, Molecular identification, Biocontrol

## Abstract

**Background:**

Studies carried out with novel 13 strains of *Trichoderma*, isolated from mangrove sediments (PE, Brazil) using morphophysiological and molecular characterization, followed evaluation of biocontrol using *Fusarium* strains isolated from Caatinga soil (PE, Brazil). *Trichoderma* strains were characterized by polyphasic taxonomic approach, and the extracted DNA was amplified with primers ITS 1 and 4, and sequenced. The biocontrol evaluation was conducted at 24 and 48 h of growth intervals by Tukey test, with a significance of 5%. Antibiosis tests were assessed in vitro by dual plate and partition plate techniques against *Fusarium* strains.

**Results:**

*Trichoderma* molecular identification, sequences of 500 bp were amplified, deposited into GenBank, and used for phylogenetic analyses. The strains were identified as *T. asperellum* (10), as *T. harzianum* (2) and one as *T. longibrachiatum*. Growth rate presented an average of 0.1207 cm h^−1^ for *Trichoderma* and lower growth rate of 0.031 cm h^−1^ for *Fusarium* spp., respectively. Antibiosis tests presented the best antagonist level of efficiency for *T. asperellum* UCP 0149 against *F. solani* UCP 1395 (82.2%) and *F. solani* UCP 1075 (70.0%), followed by *T. asperellum* UCP 0319 against *F. solani* UCP1083 (73.4%) and *T. asperellum* UCP 0168 against *F. solani* UCP1098 (71.5%), respectively.

**Conclusions:**

The data obtained in this study as tool for identification of novel *Trichoderma* strains serve as basis for development of several sustainable use for biotechnological processes. Those *Trichoderma* strains found promising for the management antagonistic potential and interaction could aid the conduct of biotechnological biocontrol of contaminants, and improve environmental conditions for the health of plants.
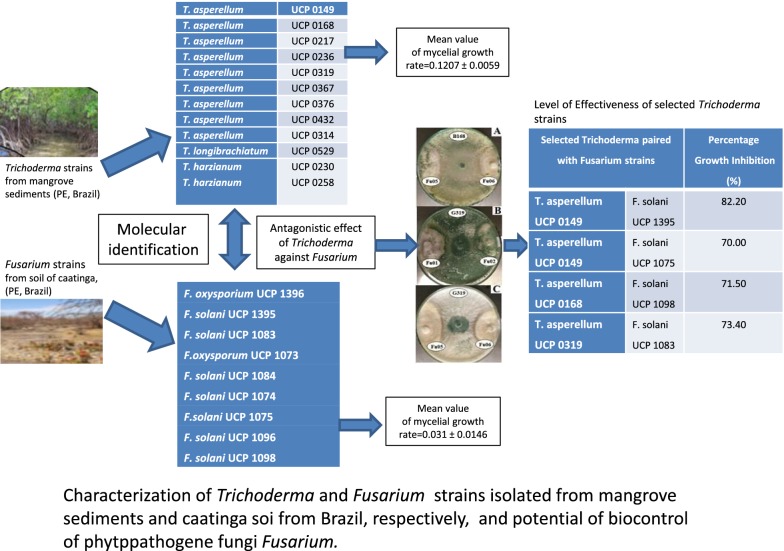

## Background

Brazil possesses 1,225,444 hectares (approximately 12,250 km^2^) of mangrove. Mangrove are considered important and transitory costal ecosystem between land environments and marine environments. These ecosystems are a dynamic ecotone between terrestrial and marine habitats, located in typical tropical and subtropical regions. They are affected by tides and of great ecological, economic, and social significance [1–3].

Approximately 90% of mangroves are distributed in South-East Asia, America, and Africa. Fungal diversity including lower fungi (oomycetes and thraustochytrids) and higher fungi (ascomycetes and basidiomycetes) are present in mangrove habitats. They are playing an important role in the nutritive cycle and support the mangrove ecosystem Important studies realized with mangrove fungi and suggest promising sources for screening new products considering the adaption of extreme conditions of the environment [4, 5].

Fungi of the genus *Trichoderma* have been isolated from estuarine environments rich in lignocellulosic materials. Their great capacity for competition and adaptation in these environments is due to: their ability to synthesize biomolecules such as enzymes and antimicrobial metabolites such as antibiotics, which are of great economic and industrial interest; their hyper parasitism; and their competition for space, oxygen, and nutrients, which has been drawing the interest of researchers [6–9].

The assays using these microorganisms, such as the species of the genus *Trichoderma*, biomolecules producers with the ability to control phytopathogens mainly in the biological and agricultural areas, resulting in formulations of regulated and commercialized products for agricultural use [10, 11]. Among these, *T. harzianum* is one of the most researched biocontrol species, followed by *T. viride, T. koningii, T. hamatum* and *T. pseudokoningii* [12].

*Trichoderma* spp. are used as biological control agents against soil-borne pathogens, such as *Fusarium*, *Pythium* and *Rhizoctonia* spp., which infect grain cultures like the French beans described in Kariuki et al. [13]. These microorganisms have been used in the management of such pathogens, especially to the export market, because they act producing antibiotics and cell wall degrading enzymes, besides acting through mechanisms of mycoparasitism and competition for nutrients and space, exercising a biological control [14–16].

However, this ability to produce fungitoxic substances can vary between species and among isolates of the same species, where some strains can produce antimicrobial metabolites while others act as promoters of plant development, making necessary a taxonomic identification among species [17, 18].

Taxonomic confirmation of species of the genus *Trichoderma*, based solely on morphological markers, can be considered limited and of low accuracy due to the plasticity of its characteristics. Therefore, molecular techniques must be combined with the adoption of a variety of parameters for correct identification of species and for phylogenetic comparisons based on target sequences, thus determining precise relationships between isolates from this genus [19, 20].

Currently, pest and disease control measures are carried out on a large scale using agrochemicals. Nonetheless, the use of such substances—besides representing onerous productive costs reflected on the price of the final products is associated to several problems, such as emergence of resistant pathogens, environmental contamination [21, 22]. Biological control stands out as an alternative for a more sustainable agricultural development, besides contributing to environmental conservation. This alternative measure has been more and more widespread on the account of being relatively simple, clean, and less onerous. In vitro tests are the basis for selecting and assessing the potential and feasibility of biocontrol of microorganisms that can prompt the growth or development of phytopathogenic agents [23, 24].

In this context, novel *Trichoderma* spp. strains were identified by polyphasic taxonomy and molecular characterization, and explored as an effective and sustainable approach for the management of biocontrol of potentially phytopathogenic *Fusarium* strains.

## Results and discussion

### Morphological identification and characterization of *Trichoderma* strains

**Fig. 1:**
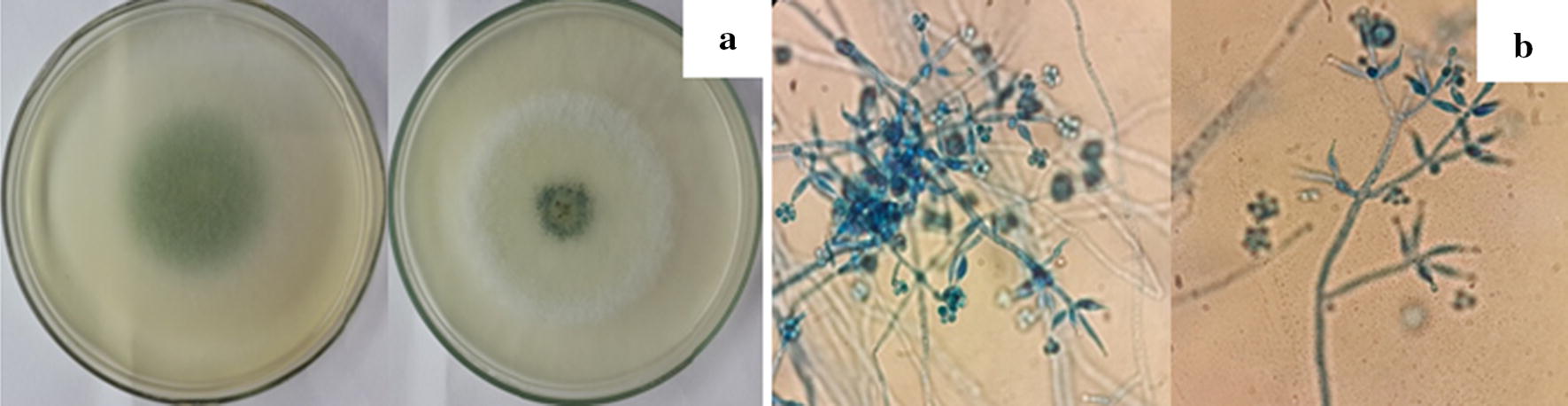
Characterization of *Trichoderma* strains by macroscopic morphology of the growth pattern of colonies in PDA medium after 5 days: **a** Optical microscopy arrangements of hyphae; **b** Conidiophores and conidia. Magnification ×400

The genus *Trichoderma* has gained immense importance since several decades due to its antagonistic ability against wide range of plant pathogens and growth promotion in crop plants. Thirteen of *Trichoderma* strains were morphologically analyzed, they shown compatible with the description for genus, respectively. They were selected and maintained in periodic cultivation on Potato Dextrose Agar (PDA) medium. Macroscopic morphology was observed in *Trichoderma* spp. strains and revealed colonies with rapid growth, concentric halos and floccose or compact surface that looked like tufts on the culture medium. The mycelium, initially of a white color, acquired green, yellow shades, or remained white, due to the abundant production of conidia. Microscopically, it was observed abundant sporulation of smooth or rough-appearance conidia, originating from branched and irregularly verticillated conidiophores, and presented conidiogenic (phialides) cells, which generally were ampliform or fusiform and arranged in clusters, according to Gam et al. [25] (Fig. 1).

Based on these characteristics, the *Trichoderma* strains could be classified in three groups, according to the identification key described by Gams and Bisset [20], Rifai [26] and Samuels et al. [27] as shown in Table 1.

**Table 1:** *Trichoderma c*lassification of thirteen strains isolated from mangrove sediments used in this study according to Gams and Bisset [20], Rifai [26] and Samuels et al. [27]

Groups	Morphology of the colony	*Trichoderma* strains Universidade Católica de Pernambuco (UCP)
Group I	In the verse the colony presented itself dark green in the center and light yellow in the borders, or fully white and reverse with no color change	*Trichoderma* sp.UCP 0149 *Trichoderma* sp. UCP 0217 *Trichoderma* sp.UCP 0258 *Trichoderma* sp.UCP 0319 *Trichoderma* sp.UCP 0367 *Trichoderma* sp. UCP 0376 *Trichoderma* sp. UCP 0314
Group II	In the verse the colony presented itself white in the center and yellow or white in the borders, and reverse with no color change	*Trichoderma* sp. UCP 0168 *Trichoderma* sp. UCP 0230 *Trichoderma* sp. UCP 0236 *Trichoderma* sp. UCP 0432 *Trichoderma* sp. UCP 0476
Group III	In the verse the colony presented itself light yellow and reverse with no color change	*Trichoderma* sp. UCP 0529

*Trichoderma* spp. showed morphological colonies, where the green color of the conidia is interleaved with the white of the mycelium, which is consistent with the characteristics previously described for this fungus using the identification key of Samuels [28] were obtained. For the strains of *Trichoderma asperellum* showed globous, subglobous to ellipsoid, and some ovoid conidia, ampuliform phialides, and presence of chlamydospores were described Samuels et al. [29].

The results obtained with morphological aspects of *Trichoderma* strains are corroborated with Samuels et al. [27] to *Trichoderma harzianum* which species presents subglobous to ellipsoid conidia, ampuliform phialides, and globous and subglobous chlamydospores and the results were corroborated with the findings of the literature [30–32].

Samuels et al. [29] and Rifai [26] describes *Trichoderma longibrachiatum* possesses subglobous to ovoid conidia and lageniform phialides. Bisset [20], still, reports the presence of yellowish-green pigment in the reverse of some cultures of this species; however, it was not observed in this work. However, the characteristics of the colony morphology serve to identify fungi of this genus *Trichoderma*, it is insufficient to distinguish the species, and is necessary to confirm those species through molecular methods [33–37]. The authors emphasizes that intraspecific differentiation is more complex as due to the overlapping of several distinctive characters, resulting in a much artificial and subjective key. The identification of the isolates in this study yielded ten species of *T. asperellum* was the most frequently sampled, two species, of which *T. harzianum*, and one as *T. longibrachiatum*. The presence of *T*. *asperellum* had already been reported in mangrove sediments from India [38].

### Molecular identification and phylogenetic analysis

**Fig. 2:**
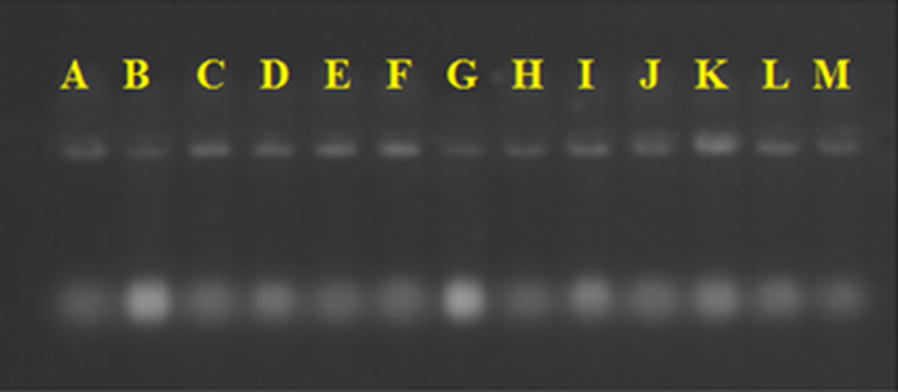
PCR amplification on 1.5% agarose gel with products obtained from the regions of ITS primers of *Trichoderma* isolates which have distinct lineage strains. *Trichoderma* isolates as mentioned in Table 1

Alexopoulos et al. [39] states that this complexity observed comes from the morphological concept of species, which is based solely on the similarities and on the characters’ discontinuity. The phylogenetic concept of species considers the genealogical, evolutionary relationships among the organisms of the group. The thirteen *Trichoderma* strains were identified by molecular level after morphological characterization using primers ITS 1 and ITS 4 amplified sequences of approximately 500 base pairs, observed on the agarose gel (Fig. 2).

Advances in the field of molecular biology, mainly in DNA analysis, enhance the way taxonomy has been done, thus justifying the performing of polyphasic approximations in studies of the different fungal groups. Using techniques of molecular identification, Hermosa et al. [40] obtained bands between 560 and 600 bp when they amplified ITS regions in order to identify 17 isolates of *Trichoderma* spp., among which are *T. harzianum*, *T. atroviride*, *T. asperellum*, *T. longibrachiatum*, used as biocontrol. Likewise, Menezes et al. [41] identified three isolates of *Trichoderma* spp. used on the biocontrol of *Fusarium*. The results described by these authors are similar to those found in this work, where primers ITS 1 and ITS 4 amplified sequences of approximately 500 base pairs for the studied strains of the genus *Trichoderma*.

The sequences, obtained from the sequencing, were assembled by the use of Staden package [42]. For close species, the sequences were compared to the other available on GenBank, using the BLAST tool available on the National Center for Biotechnology Information (NCBI). Multiples alignments of the sequences were estimated using Muscle algorithm in MEGA v.7 and adjusted manually when necessary. For multilocus analysis, the loci were concatenated by the use of Sequence Matrix v.1.8 [43, 44]. The sequences’ access codes of deposit on GenBank are in Table 2.

**Table 2:** *Trichoderma* strains identification, culture collection number, isolation from, country and number of the genetic sequences of the ITS region

Taxon	Collection number	Isolation from	Country	Number NCBI/ITS
*T. asperellum*	CBS 433.97^c^	Soil	USA	AY380912
*T. asperellum*	IBSD T39	Soil	India	JX518901
*T. asperellum* ^a^	UCP 0149	Mangrove sediments	Brazil	MF974884
*T. asperellum* ^a^	UCP 0168	Mangrove sediments	Brazil	MF974875
*T. asperellum* ^a^	UCP 0217	Mangrove sediments	Brazil	MF974876
*T. asperellum* ^a^	UCP 0236	Mangrove sediments	Brazil	MF974877
*T. asperellum* ^a^	UCP 0319	Mangrove sediments	Brazil	MF974878
*T. asperellum* ^a^	UCP 0367	Mangrove sediments	Brazil	MF974879
*T. asperellum* ^a^	UCP 0376	Mangrove sediments	Brazil	MF974880
*T. asperellum* ^a^	UCP 0432	Mangrove sediments	Brazil	MF974881
*T. asperellum* ^a^	UCP 0314	Mangrove sediments	Brazil	MF974883
*T. asperellum* ^a^	UCP 0476	Mangrove sediments	Brazil	MF974882
*T. brunneoviride* ^b^	CBS121130^c^	–	Germany	EU518659
*T. longibrachiatum*	CBS 816.68^c^	–	USA	EU401556
*T. longibrachiatum*	NRRL 54514	*Gloeophyllum trabeum*	USA	HQ882796
*T. longibrachiatum* ^a^	UCP 0529	Mangrove sediments	Brazil	MF974874
*T. harzianum*	CBS 226.95^c^	Soil	UK	AJ222720
*T. harzianum*	CBS 227.95	Soil	UK	AJ222721
*T. harzianum* ^a^	UCP 0230	Mangrove sediments	Brazil	MF974886
*T. harzianum* ^a^	UCP 0258	Mangrove sediments	Brazil	MF974885

CBS: Centraalbureau voor Schimmelcultures, Utrecht, The Netherlands; IBSD: Institute of Bioresources and Sustainable Development, Manipur, India; NRRL: Agricultural Research Service Culture Collection, Peoria, USA.; UCP: Universidade Católica de Pernambuco

^a^Taxon in bold were found in this study. ITS: Internal transcribed spacer (ITS1-5.8S-ITS2)

^b^Outgroup isolate

^c^*Type* species culture

Among 13 *Trichoderma* strains analyzed, ten strains were identified as *T. asperellum* UCP 0149, following codifications UCP 0168, UCP 0217, UCP 0236, UCP 0319, UCP 0367, UCP 0376, UCP0432, UCP 0314 and UCP 0476; two strains as *T. harzianum* UCP 0230 and UCP 0258, and strain *T. longibrachiatum* UCP 0529 (Table 2).

**Fig. 3:**
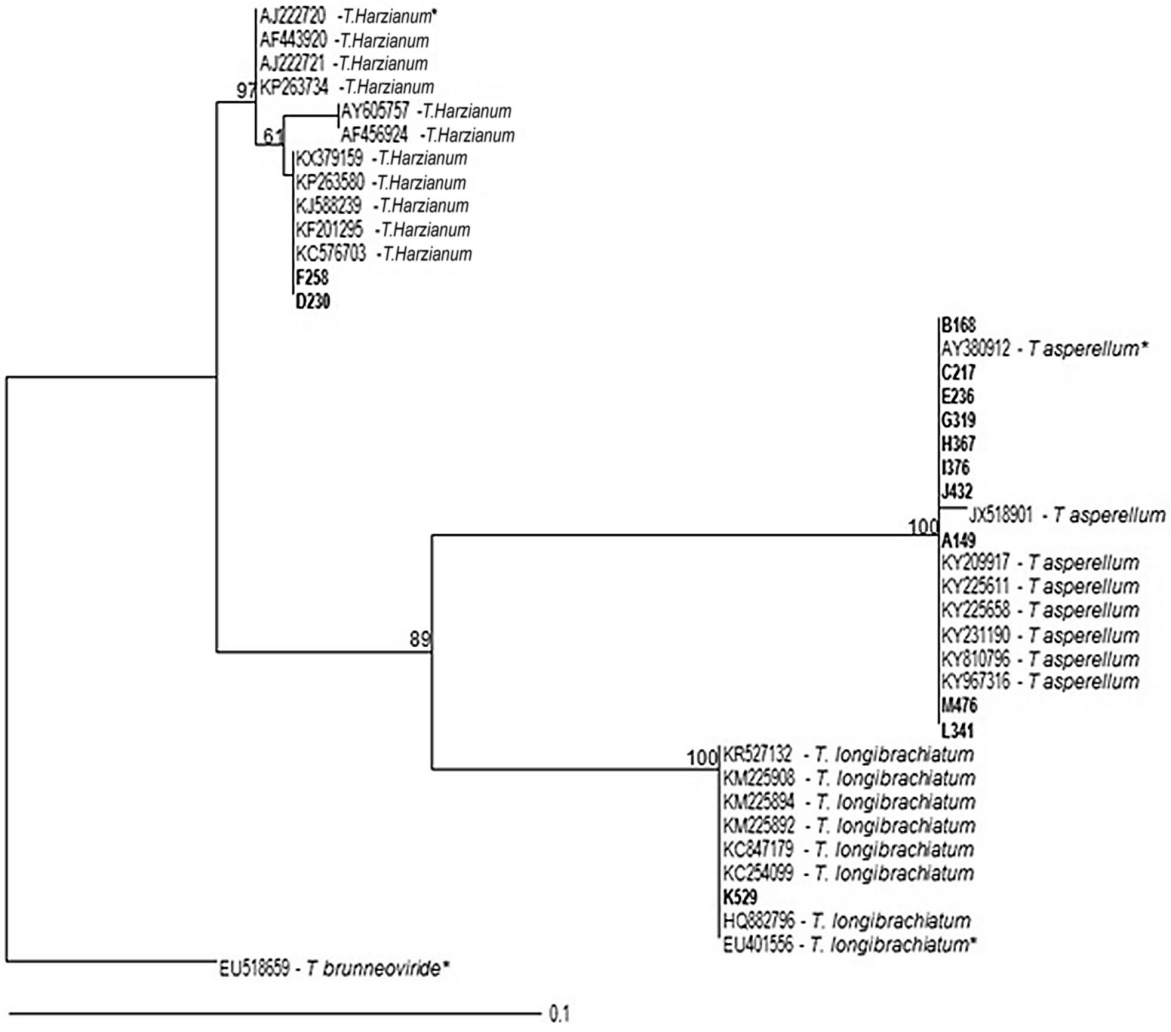
Phylogenetic analysis of maximum likelihood tree was constructed by 602 bp aligned with partial sequences of the Internal transcribed spacer—ITS (ITS1, 5.8 S and ITS2), with 19 taxa belonging to the *Trichoderma* species, compatible with the isolates examined in this research

All sequences were used for the formation of the phylogenetic tree, along with sequences of species types. The alignment and phylogenetic analysis of these sequences showed groupings that corroborate the taxonomic identification of the species studied in this work, presenting 602 characters, of which 68 were informative, 94 were variable and 506 were constant. The maximum likelihood analysis generated a consistent topology tree, in which three groups corresponding to the genus *Trichoderma* were observed, namely: *T. harzianum*, *T. longibrachiatum* and *T. asperellum,* which showed ramifications among the individuals of the other clades, as verified in Fig. 3.

The species chosen as outgroup was *T. brunneoviride*, for despite being within the genus, it possesses a nucleotide sequence that diverges on other clades members, occupying a base position and differing from other groupings [45–47].

The isolates UCP 0230 and UCP 0258 were grouped in the *T. harzianum* section with the England isolate type, forming a clade with bootstrap values of 85%. When related to each other, they formed a clade with bootstrap values of 99% (Fig. 3). In the T. *longibrachiatum* section, the UCP 0529 isolate formed a clade with bootstrap values of 100% with the isolate type of the United States of America (USA). The *T. asperellum* section comprised the remaining isolates in two subclades with 100% bootstrap values, where *T. asperellum* UCP 0376, UCP 0314 and UCP 0432 were grouped with the India rate and *T. asperellum* UCP 0476, UCP 0319, UCP 0236, UCP 0168, UCP 0367, UCP 0149, UCP 0217 with the USA type rate. However, the clade formed a subgroup with bootstrap values of 90% when compared to the isolate from the *T. longibrachiatum* section. This indicates a probable speciation event between these two species, thus forming a consistent grouping within the section. Bootstrap values above 70% (indicating the ML/MP ratios) are shown near the nodes. Isolates from this study were written in bold in the highlighted branch; *Trichoderma brunneoviride*, isolate CBS121130, was used as outgroup, and taxa typed with NCBI access code followed by (*) represents the type of species (Fig. 3).

On the purpose of establishing the relationship among genetic proximity, phylogeny and antagonistic activity against possible phytopathogens of the genus *Fusarium*, and of eliminating possible duplicities of such isolates, it was carried out a sequencing of the genomic DNA of *Trichoderma* strains selected for this work. Tests with such goal were also conducted by Colonia and Chagas Junior [48] using ITS region sequencing in the identification of the strains of *Trichoderma* spp. isolated from *Trichoplus* JCO fertilizer.

Results from the phylogenetic groupings showed that the strains of *Trichoderma* used in this work formed a reliable maximum likelihood tree, where support values—verified in each group—and the taxonomic inferences attributed to the individuals—in accordance with the respective grouping sections—are adequate, according to Kubicek et al. [49].

In this study, strains of *T. harzianum* formed a consistent cluster supported by a bootstrap value of 85%. However, the subdivision observed in this clade, that separates isolates UCP 0230/UCP 0258 from CBS 226.95 and CBS 227.95 (Fig. 3), can be related to recent speciation phenomena or genetic variability present in this species. For Druzhinina et al. [50], *T. harzianum* is one of the species with most variability and, nowadays, is considered a species complex.

The species chosen as outgroup was *T. Brunneoviride* because, despite being within the genus, it has a nucleotides sequence that diverges in the other clades individuals. Besides, it occupies a base position that differs from other groups [47, 51].

Studies carried out by Hoyos-Carvajal et al. [32] indicated the predominance of *T. asperellum* species, followed by *T. harzianum*, in the biodiversity of the neotropics, what can be explained by the high intraspecific genetic variability, colonization ability and high levels of sporulations in different substrates and carbon sources. In this work, it was also verified the predominance of *T. asperellum* species, followed by *T. harzianum*, among the samples of the genus *Trichoderma* selected and identified by molecular techniques.

Usage of molecular tools for species identification based on DNA sequences has been showing that *Trichoderma* species structured on classic concepts are, in fact, composed of two or more phylogenetic species (aggregate species), which can exhibit specific or global ecological niches [52]. The species *T. harzianum* is been recognized as global species, colonizing many substrates and ecological niches [53].

### Evaluation of mycelial growth rate of *Trichoderma* and *Fusarium* strains

All *Trichoderma* strains showed a similar in vitro growth pattern, with an average growth rate of 0.1207 cm h^−1^. However, *Fusarium* spp. growth rates showed the media of 0.031 cm h^−1^. This can be considered significant when compared to the mean deviation of 0.0146 cm h^−1^, which represents a variation of approximately half of this average. The growth rates of *Trichoderma* spp. were approximately four times greater than the *Fusarium* strains growth rate after a 72 h period of evaluation (Table 3).

**Table 3:** In vitro colony diameter and mycelial growth rate of *Trichoderma* spp. and *Fusarium* strains in the 48 h interval

*Trichoderma* strains	DG (cm)		MGR	*Fusarium* strains	DG (cm)		MGR
	24 h	48 h			24 h	48 h	
*T. asperellum* UCP 0149	1.9	4.7	0.11	*F. oxysporum* UCP 1396	1.1	2.4	0.05
*T. asperellum* UCP 0168	2.0	5.2	0.13	*F. oxysporum* UCP 1073	0.7	2.1	0.05
*T. asperellum* UCP 0217	2.2	5.2	0.12	*F. solani* UCP 1083	0.7	1.1	0.01
*T. harzianum* UCP 0230	1.5	4.0	0.10	*F. solani* UCP 1395	0.8	1.3	0.02
*T. asperellum* UCP 0236	2.0	5.0	0.12	*F. solani* UCP 1084	0.8	1.9	0.04
*T. harzianum* UCP 0258	2.0	5.0	0.12	*F. solani* UCP 1074	0.7	1.3	0.02
*T. asperellum* UCP 0319	1.7	4.8	0.12	*F.solani* UCP 1075	0.6	1.3	0.02
*T. asperellum* UCP 0367	1.3	5.0	0.15	*F. solani* UCP 1096	0.6	1.1	0.02
*T. asperellum* UCP 0376	1.3	4.5	0.12	*F. solani* UCP 1098	0.7	2.0	0.05
*T. asperellum* UCP 0432	2.0	5.0	0.12				0.031 ± 0.0146^a^
*T. asperellum* UCP 0314	1.5	4.5	0.12				
*T. asperellum* UCP 0476	2.0	5.0	0.12				
*T. longibrachiatum* UCP 0529	1.2	4.3	0.12				
			0.1207 ± 0.0059^a^				

DG: in vitro colony diameter growth (cm); MGR: mycelial growth rate (cm h^−1^)

^a^Mean values of mycelial growth rate ± mean deviation values

### Phenomenon of antibiosis of *Trichoderma* against *Fusarium* strains

The biocontrol potential by pairing test of *Trichoderma* strains cultures against the strains of *Fusarium* showed that all the strains of *Trichoderma* presented biocontrol action, affecting the development pattern of *Fusarium* spp. colonies. *Trichoderma* colonies presented a faster growth in the plates of paired cultures, being capable of growing on the potential pathogens and, thus, preventing their mycelial development by nutrient and space competition. Moreover, it was verified the inhibition by way of production of secondary metabolites, which demonstrated the capacity for biocontrol interaction through more than one mechanism [54].

**Fig. 4:**
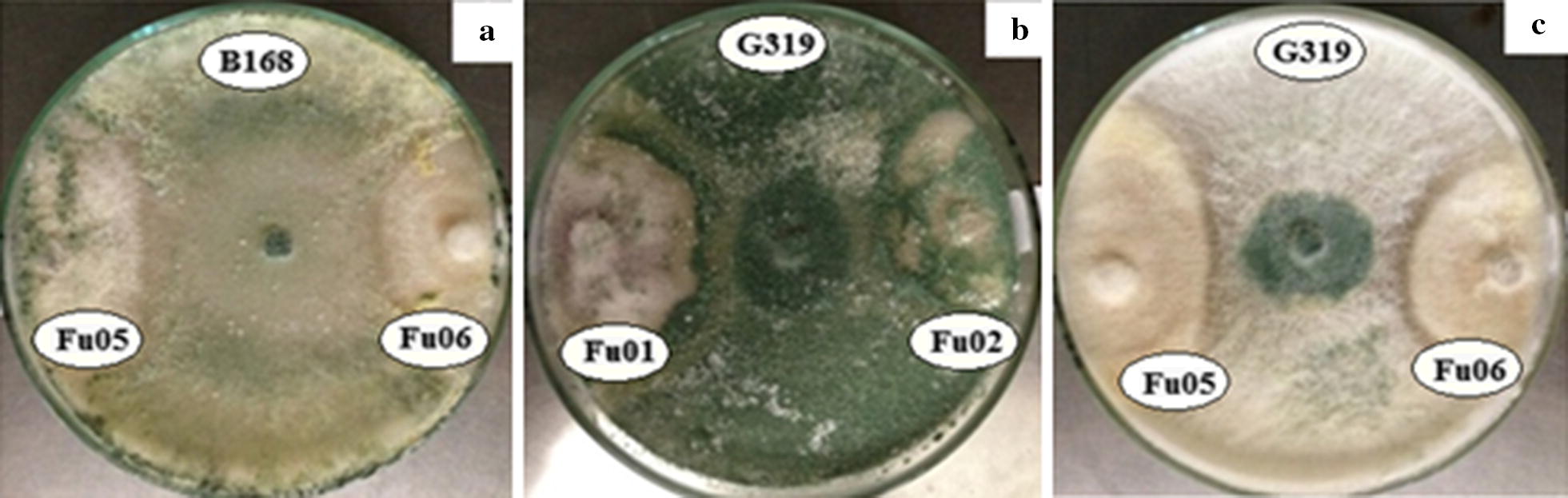
Antagonistic effect through mycoparasitism by the overlapping of colonies (**a**) and by formation of inhibition halo (**b**, **c**) of *Trichoderma* spp. colonies inhibiting the growth of *Fusarium* spp. strains in direct confrontation in vitro. Isolates *F. oxysporum* UCP 1396 and *F. solani* UCP 1074 under effect of *T. asperellum* UCP 0319 (**a**, **b**). Isolates *F. solani* UCP 1395 under effect of *T. asperellum* UCP 0314 (**c**)

It was verified that six *Trichoderma* spp. strains reduced down to 50% the growth of *Fusarium* spp. strains. The strain of *T. asperellum* UCP 0319 presented two distinct behaviors when paired with different isolates of the genus *Fusarium* (Fig. 4a, b). The analysis indicated this strain’s effect through mycoparasitism when the antagonist removes nutrients from the parasitical fungi hyphae causing their death, and through competition (Fig. 4a) against the strain of *F. oxysporum* UCP 1396. The inhibition halo formation can be observed in Fig. 4b against *F. solani* UCP 1074, as well as against the strains of *F. solani* UCP 1084, UCP 1395 and UCP 1096. This behavior was also observed in the strains *T. asperellum* UCP 0217, UCP 0258, UCP 0367 and UCP 0432, and *T. harzianum* UCP 0230. The analysis of variance, in a factorial arrangement by Tukey’s test of 5% probability, the percentages of inhibition of the possible *Fusarium* pathogenic strains were significant to different strains of *Trichoderma* (p ≤ 0.01).

### Level of effectiveness of *Trichoderma* against *Fusarium* strains

The percentages of mycelial growth inhibition profile (GIP—%) of *Trichoderma* strains were analyzed by analysis of statistical variance, with significant variation (p ≤ 0.05), when paired were against *Fusarium* strains variated of 50 to 82.2% of inhibition.

The best antagonist phenomenon of effectiveness was observed to *T. asperellum* UCP 0149, presenting the highest inhibitory percentage (82.2% inhibition) against *Fusarium solani* UCP 1395, regarding the others samples analyzed in this work. The statistical analysis showed percentage inhibition of *F. solani* UCP 1083 by *T. asperellum* UCP 0319 stood out as the second antagonist effectiveness for this strain, with a percentage inhibition of 73.4%, respectively.

**Fig. 5:**
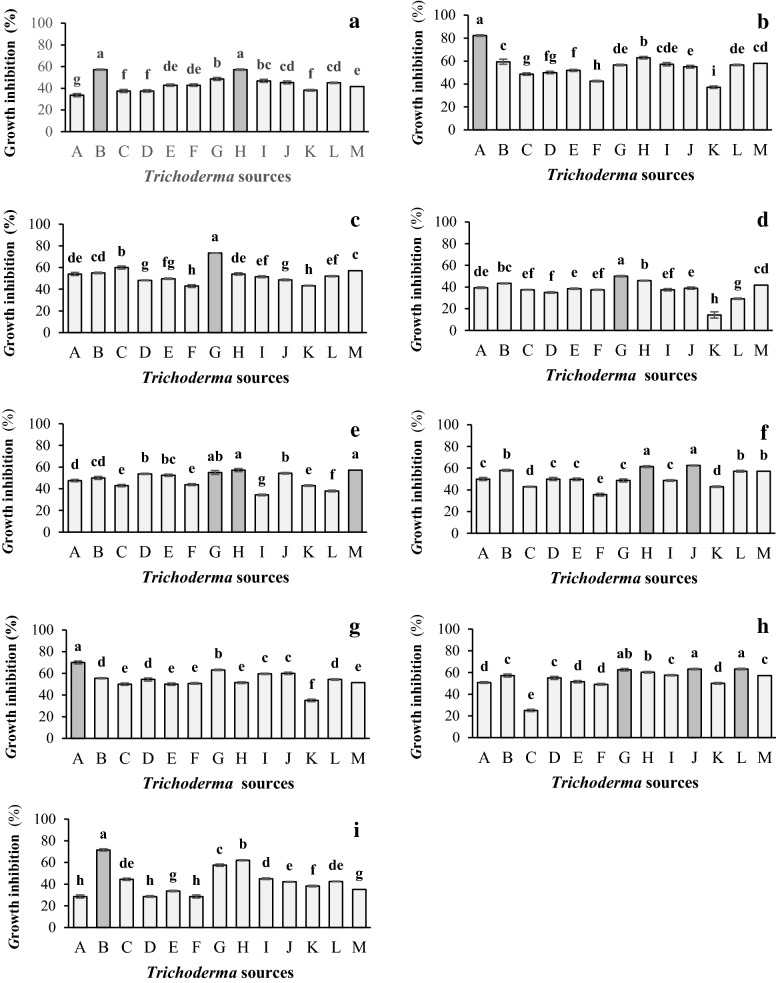
Antagonistic effect through mycoparasitism due to the overlapping of colonies (**a**) and due to the formation of inhibition halos (**b**, **c**) of *Trichoderma* spp. Colonies, thus inhibiting the growth of *Fusarium* spp. strains in direct confrontation *in vitro*. Isolates *F. oxysporium* UCP 1396 and *F. solani* UCP 1074 under effect of *T. asperellum* UCP 0319 (**a**, **b**). Isolates *F. solani* UCP 1395 under effect of *T. asperellum* UCP 0314 (**c**)

In the light of such results, it can be observed that strains of *F. oxysporum* are more resistant to the antibiosis action of *Trichoderma* strains regarding the *F. solani*. Among the strains of *T. asperellum* were significantly better antagonists than the strains of *T. harzianum* and *T. longibrachiatum*, and showed percentage of inhibition equal or superior to 50% in the most associations. The effective levels of antagonism, according to Sangoyomi [55] and described by Okigbo and Emeka [57], all strains of *T. asperellum* UCP 0149, UCP 0168, UCP 0432, UCP 0367 and UCP 0319 described in these study, and promoting inhibition over 60% in at least two distinct isolates of *Fusarium* strains, as well as strains *T. asperellum* UCP 0314, *T. asperellum* UCP 0217 and UCP 0376, with the same percentage inhibition in at least one strain of *Fusarium* (Fig. 5).

The genus *Trichoderma* is universally recognized by its biotechnological potential in agriculture—as in biocontrol—as well as in industrial applications, in production of enzymes. Therefore, it is a sustainable alternative to chemical pesticides, presenting itself more trustworthy for animal health, ecologically safer, and economically viable [58].

All strains of *Trichoderma*, isolated from mangrove sediments, reduced the mycelial growth of all potentially pathogenic *Fusarium* strains, isolated from caatinga soil, tested in this work. The different *Trichoderma* species showed variable levels of growth rate and antagonism in regards to the *Fusarium* species in the tests of culture pairing. It was not observed the growth of the probable pathogen on colonies of *Trichoderma*; most of the isolates were graded 2 according to Bell scale. This fact corroborates the works conducted by Louzada et al. [59], on which the antagonistic potential was tested against *S. sclerotiorum* and *F. solani*.

The specificity of biocontrol agents against phytopathogenic agents was evidenced by Vinale et al. [7], Kariuki et al. [13] and Zeilinger et al. [60], recognized *Trichoderma* spp. as producers of various cell wall degrading enzymes and of secondary metabolites with biocontrol action.

The new strains of *T. asperellum* UCP 0149, UCP 0168, UCP 0432, UCP 0314, UCP 0319, UCP 0376, UCP 0217 and UCP 0367 identified in this work presented better biocontrol performance, inhibiting the growth of all strains of *F. solani* UCP 1395, UCP 1075, UCP 1098, UCP 1074, UCP 1096, UCP 1074, UCP 1083 and of *F. oxysporum* UCP 1396 and UCP 1073, when compared to *T. harzianum* and *T. longibrachiatum*. In the present study, it is deduced that the inhibitory actions of the studied strains of *Trichoderma* are possibly linked to the antibiosis action through the formation of inhibitory halo, hyperparasitism and competition (Tables 2, 3 and 4).

**Table 4:** Biocontrol potential of *Trichoderma* spp. strains against *Fusarium* spp. strains according to Bell scale [56]

*Trichoderma* strains	*Fusarium* strains^a^								
	F1	F2	F3	F4	F5	F6	F7	F8	F9
*T. asperellum* UCP 0149	3.92	1.61^b^	2.44	3.35	2.78	2.64	1.89^b^	2.6	3.57
*T. asperellum* UCP 0168	2.31	2.22^b^	2.4	3.04	2.64	2.28	2.38	2.31	1.85^b^
*T. asperellum* UCP 0217	3.52	2.72	2.2^b^	3.52	3.08	3.08	2.64	3.75	2.97
*T. harzianum* UCP 0230	3.52	2.64	2.74	3.77	2.46	2.64	2.43	2.4	3.57
*T. asperellum* UCP 0236	3.08	2.55	2.65	3.43	2.51	2.65	2.64	2.56	3.92
*T. harzianum* UCP 0258	3.08	3.11	3.08	3.52	3.01	3.71	2.6	2.69	3.57
*T. asperellum* UCP 0319	2.72	2.33	1.8^b^	2.64	2.4	2.72	2.09^b^	2.11^b^	2.3
*T. asperellum* UCP 0367	2.31	2.1^b^	2.44	2.88	2.31	2.15^b^	2.57	2.19^b^	2.13^b^
*T. asperellum* UCP 0376	2.82	2.31	2.56	3.52	3.85	2.72	2.22^b^	2.3^b^	2.93
*T. asperellum* UCP 0432	2.91	2.4	2.72	3.38	2.43	2.11^b^	2.2^b^	2.09^b^	3.13
*T. longibrachiatum* UCP 0529	3.46	3.55	3.05	4.29	3.08	3.08	3.77	2.64	3.46
*T. asperellum* UCP 0314	2.93	2.33	2.54	3.54	3.48	2.31	2.43	2.09^b^	3.11
*T. asperellum* UCP 0476	3.17	2.28	2.31	3.15	2.31	2.31	2.56	2.31	3.75

Okigbo and Emeka [55], Bell et. al. [56] and described by Sangoyomi [57]

^a^Strains of *Fusarium* spp. identified on the horizontal axis by the letters: *Fusarium oxysporum* UCP 1396 (F1); *F. solani* UCP 1395 (F2); UCP 1083 (F3); *F. oxysporum* UCP 1073 (F4); UCP 1084 (F5); UCP 1074 (F6); UCP 1075 (F7); UCP 1096 (F8); and UCP 1098 (F9), in joint culture with thirteen isolates of *Trichoderma* spp. identified on the vertical axis

^b^Effective inhibition of strains of *Fusarium* spp

The strains of *F. oxysporum* presented themselves more resistant to the antibiosis mechanisms when compared to the strains of *F. solani*. The general average inhibition observed was superior to 50% and the best results were connected to values above 70%, indicating that each species of *Trichoderma* possesses different abilities to inhibit different strains of *Fusarium* (Table 4) [1, 58, 61, 62].

Effective results against isolates *F. oxysporum* in pepper cultivars were also obtained by Hernández Castillo et al. [63] with the use of isolates of *Trichoderma asperellum*, obtaining an inhibition of 86.5%. According to the authors, the metabolites produced by *T. asperellum* have potential to reduce the reproductive capacity of *F. oxysporum*, diminishing sporulation and inhibiting conidia germination.

Different results were observed by Taribuka et al. [64] on selecting strains of *Trichoderma* that are potentially antagonistic to *F. oxysporum*. The highest percentages inhibition of growth were provided by *T. gamsii* (60.61%), followed by *T. harzianum.*psr-1 (59.08%), *T. harzianum*.swn-1 (55.80%), *T. koningiopsis* (55.58%), *T. harzianum*.swn-2 (54.05%) and *T. asperellum* (49.67%).

Results obtained in the present study, in antibiosis tests, presented variations regarding the degree of antagonism, where not all isolates of a same species of the genus *Trichoderma* were able to inhibit with the same effectiveness the same tested *Fusarium* strain. This fact may be indicative of the existence of specificity between the antagonist and the potential phytopathogen, suggesting the involvement of various genes and genetic factors interacting with the environment.

According to Perello et al. [65], more than one mechanism of action might be simultaneously involved in antagonistic actions. Louzada et al. [59] states that not all isolates of a same species of the biocontrol agent are capable of exercising hyperparasitism, suppressing the pathogen growth through other mechanisms like antibiosis or competition. According to Howell [66], species of *Trichoderma* have the ability of suppressing the growth of various fungi—in solid culture mediums—possibly linked to antibiosis action, hyperparasitism and competition.

Chen and Zhuang [62] states that species of *Trichoderma* grow faster because they use the food source in a more efficient way. In this study, the strains of *Trichoderma* not only presented the highest growth rates in regards to the strains of *Fusarium*, but they significantly exceeded the values of about 0.33 mm h^−1^ indicated by Moretto et al. [67]. An agent of biological control can excrete one or more metabolites that slow or inhibits the growth of pathogenic agents in the surrounding area. This phenomenon is called antibiosis [59, 65]. Works developed by Howell [68] prove the production of cell wall degrading enzymes, like chitinases and cellulases, as means of action of *Trichoderma* spp. against phytopathogenic fungi. According to Bosah et al. [68], the paired culture test is extremely important in the area of biological control of phytopathogens, because a good performance in this test indicates that the antagonist agent is an effective biocontroller.

The strains of *T. asperellum* UCP 0149, *T. asperellum* UCP 0168 and *T. asperellum* UCP 0319 presented the best antagonistic effect on the growth of *Fusarium* strains, on the observation of the inhibition zone between colonies of both fungi or on the overlapping of the mycelium of *Trichoderma* in the *Fusarium* colony (Fig. 4). Observations of such nature are used to evaluate the antagonistic potential of *Trichoderma* spp. and the obtained results prove that the studied strains have the potential to suppress the growth of *Fusarium* species, corroborating the findings of Taribuka and Bae [64], respectively.

The species of *Trichoderma* detect and locate the mycelium of susceptible fungi and grow on its direction, because they respond to the chemical stimuli produced by the host fungus. Besides, competition is one of the main characteristics of *Trichoderma* isolates due to their high mycelial growth rate [67], as proved in this work. In this context *Trichoderma* based emerges as an ecologically attractive alternative as biocontrol agent to phytopathogens considering the results obtained suggest the use as commercial products considering the effectiveness level of *T. asperellum* UCP 0149 against *F. solani* UCP 1395.

## Conclusions

It was shown that, in the mangrove sediment of a Brazilian ecosystem, there are fungi of the novel *Trichoderma* strains which vary greatly from each other. It is also important to have greater knowledge of the ecology of these species and their responses to environmental or anthropogenic disturbances which may interfere with the equilibrium of these ecosystems. We studied a wide range of morphological and defined phylogenetic lineages based on their morphological characters. The results suggested that *T. asperellum, T. harzianum* and *T. longibrachiatum* fungal strains exhibit heterogeneity in genome structure of DNA sequence and similarity of ITS1 and four sequences in most taxa. *Trichoderma* strains showed a capacity for inhibiting mycelia growth of seven strains of *Fusarium solani* and two strains of *F. oxysporum*, all of which were isolated from caatinga soil of Brazil. However, only three selected strains identified as *T. asperellum* showed the best antagonist results in order to achieve the highest level of effectiveness and the possibility of applying them in an eco-friendly way and at low cost as biological agents against phytopathogenic *Fusarium* strains, and in addition, as target specific agents when compared with synthetic fungicides. Moreover, studies of those filamentous fungi which have a good potential for antagonistic interaction can both aid the conduct of biotechnological processes, and improve environmental conditions and there the health of plants.

## Materials and methods

### Microorganism and culture conditions

Thirteen *Trichoderma* and Nine Fusarium strains were kindly released to the researchers from the UCP (Universidade Católica de Pernambuco) Culture Collection, which is registered in the WFCC (World Federation for Culture Collection). The strains of Fusarium were isolated from Caatinga soil in Serra Talhada, Pernambuco, Brazil; and *Trichoderma* spp. were obtained from mangrove sediments of Rio Formoso, Pernambuco, Brazil, and maintained on Sabouraud dextrose agar at 5 °C.

### Morphological characterization

Morphological identification was accomplished through classification keys of Gams and Bisset [20], Rifai [26] and Samuels et al. [27], taking into consideration macroscopic characteristics like color and texture of the colony surface verse and reverse, presence or absence of pigmentation, and pattern of growth and sporulation. Microscopic characters were analyzed according to morphology, size and disposition of the conidia and the phialides, using slides prepared by the microculture in EMA technique. The material was then dyed with cotton blue so that it could be visualized. Growth rate and morphological characteristics were analyzed in PDA and malt extract (EMA) (Himedia).

### Extraction and amplification of genomic DNA

Thirteen samples of *Trichoderma* spp. underwent species-level molecular identification and phylogenetic analysis. Genomic DNA was extracted from 7 day old mycelial growth at 25 °C by the method adapted from Murray and Thompson [69]. Sequences of internal transcribed spacer (ITS) Regions 1 and 2, including the 5.8S, were amplified using primers ITS 1 (TCC GTA GGT GAA CCT GCG G) and ITS 4 (TCC TCC GCT TAT TGA TAT GC). Each 25 μl of the polymerase chain reaction (PCR) mix included: 13.85 μl ultrapure water, 1 μl template DNA, 1.5 μl of each primer (10 μM, synthesized by Invitrogen-Carlsbad, CA), 2.5 μl of dNTP mix and 4.63 μl of Taq DNA polymerase mix (0.05 μl-1 Taq DNA polymerase, reaction buffer, 4 mM MgCl2, Thermo Scientific, Waltham, USA). PCR reactions were performed in a SimpliAmp™ Thermal Cycler (applied biosystems) at 94 °C for 5 min, followed by 35 cycles at 94 °C for 1 min (denaturation), 57 °C for 1 min.

### Analysis of the products obtained by PCR

To verify the efficiency of the PCR reaction, 3 μl of the substances obtained were stained with 3 μl of SYBR^®^ Green dye (Thermo Scientific, Waltham, USA) and analyzed by 1.0% agarose gel electrophoresis in 0.5× TBE buffer (Tris–borate–EDTA 100 mM Tris base and 2.0 mM EDTA solution pH 8.0) and 50 mM of boric acid. Electrophoresis occurred at 75v for a period of 40 min. After the run, the gels were revealed and visualized under an ultraviolet transilluminator to check the amplification and purity, and then were photographed for documentation purposes [69, 70]. The amplicons were purified and sequenced by Macrogen Inc., Korea (http://www.127macrogen.com). Nucleotide sequences obtained were checked and edited using Staden Package 2.0 software packages. Subsequently, the consensus sequences obtained in this study were compared with other using Molecular Evolutionary Genetics Analysis (Mega BLAST) tool and deposited into GenBank databse (http://www.ncbi.nlm.nih.gov).

### Molecular identification and phylogeny

Nucleotides sequences obtained were checked and edited with Staden Package 2.0 software packages. Subsequently, the consensus sequences were compared to the Lasiodiplodia ex-type reference sequences, retrieved from GenBank using Mega BLAST, and deposited into GenBank database (http://www.ncbi.nlm.nih.gov). Nineteen taxa belonging to the *Trichoderma* species, compatible with the isolates of this study, were used to construct the phylogenetic tree, with *Trichoderma brunneoviride* CBS121130 used as outgroup and taxa written with NCBI access code and followed by (*) representing the type species. The phylogeny was inferred by comparing locus ITS individual phylogenetic groupings through Analysis of Maximum Likelihood Estimates (MLE) on CIPRES portal (http://www.phylo.org). MLE analyses were estimated with RAxML-HPC2 on XSEDE, model GTR GAMMA, and the best topology tree related to the Bootstrap values in 1000 pseudoreplicates. Only sequences that obtained published results—and that were searched on BLASTN—were used, with high score (1000) and e-value equal to zero.

### Determination of growth rates *Trichoderma* spp. and *Fusarium* strains

In order to evaluate growth rates, each isolate of *Trichoderma* spp. and *Fusarium* strains were cultured individually for 5 days at 25 °C in PDA medium. From this culture, disks (0.6 cm in diameter) of fungal structures were deposited in the center of a Petri dish containing PDA. These dishes were incubated at 25 °C with a 12 h photoperiod. After 24 and 48 h of incubation, the diameters (cm h^−1^) of colonies were measured in two perpendicular directions. Growth rates were determined according to the equation: G_R_ = (G_2_ − G_1_)/(T_2_ − T_1_); in which: G_R_ = growth rate; G_1_ = growth after 24 h; G_2_ = growth after 48 h; T_1_ = 24 h; T_2_ = 48 h [67].

### Antagonist determination effect of *Trichoderma* spp. against *Fusarium* strains

To analyze the antagonistic action of the isolates of *Trichoderma* spp. on the samples of *Fusarium* strains, the technique used was direct confrontation in vitro, using the method of culture pairing in Petri dishes. Following Moretto et al. [67], for each *Fusarium* strains, discs (6 mm diameter) containing fungal structures were deposited at one end of the Petri dish containing PDA medium (approximately 1 cm from the end of the plate). After 72 h, a disk of *Trichoderma* spp., with 5 days of growth, was deposited 3.5 cm away from the colony of the possible phytopathogen. The design was entirely randomized with four replicates. Control was represented by the *Fusarium* strains samples without the presence of the antagonist and by the *Trichoderma* samples without the presence of the probable phytopathogen. The plates were maintained at 25 °C with a 12 h photoperiod for 6 days. After this period, the growth of colonies of *Fusarium* strains was checked. In accordance with the methodology of Camporota [71], the percentage of colonization (%C) of each antagonist isolate was calculated using the formula: %C = (DT/DE) × 100, where DT is the distance between colonies after mycelial growth stabilizes and DE is initial distance between the two mycelial discs. The inhibition index of *Fusarium* in relation to *Trichoderma* was determined by the relation I = 100 − %C. In addition to the  %C values, each *Fusarium* isolate was classified as per the degree of antagonism (G), according to a scale of notes by Bell et al. [40], shown in Table 5. The percent growth inhibition was determined as a guide in selecting the degree of effectiveness in the control of *Fusarium* spp. samples for the three treatments.

**Table 5:** Classification of the degree of antagonism (G), according to the scale of Bell et al. [56]

Colonization pattern	Degree of antagonism (G)
Biocontrol agent grows completely over the pathogen, covering the entire surface of the culture medium	1
Biocontrol agent grows to at least about 2/3 of the surface of the culture medium	2
Biocontrol agent and pathogen colonize approximately half the surface of the culture medium (more than 1/3 and less than 2/3) and neither appears dominating the other	3
Pathogen colonizes at least 2/3 of the surface of the culture medium and exhibits resistance to the biocontrol agent	4
Pathogen grows completely on the biocontrol agent and occupies the entire surface of the culture medium	5

### Statistical analysis

Differences of the antagonistic action of *Trichoderma* isolates over *Fusarium* were determined by factorial design in ANOVA and the means were compared by Tukey test at 5% significance using the ASSISTAT^®^ program.

## Highlights

- We have focused upon *Trichoderma*-mediated fungal taxonomy, biodiversity and antibiosis against to *Fusarium* strains.
- Significant presence of *Trichoderma asperellum*, followed *T.harzianum* and *T. longibrachiatum* were detected from mangrove sediments.
- *Trichoderma asperellum* strains showed their efficiency as biocontrol agent of phytopathogenes *Fusarium solani*.

## Abbreviations

BOD: biochemical oxygen demand
CBS: Centraal bureau voor Schimmelcultures, Utrecht, The Netherlands
DMSO: dimethyl sulfoxide
DNA: deoxyribonucleic acid
dNTP: nucleoside triphosphates containing deoxyribose
EDTA: ethylene diamine tetra acetic acid
PDA: Potato Dextrose Agar
GIP: growth inhibition profile
IBSD: Institute of Bioresources and Sustainable Development, Manipur, India
ITS: internal transcribed spacer
MgCl: magnesium chloride
MLE: Maximum Likelihood Estimates
NCBI: National Center for Biotechnology Information
NRRL: Agricultural Research Service Culture Collection, Peoria, USA
PCR: polymerase chain reaction
UCP: Universidade Católica de Pernambuco
UK: United Kingdom
USA: United States of America
WFCC: World Federation for Culture Collection
